# C1q/TNF-related protein 4 restores leptin sensitivity by downregulating NF-κB signaling and microglial activation

**DOI:** 10.1186/s12974-021-02167-2

**Published:** 2021-07-18

**Authors:** Liu Ye, Gongwei Jia, Yuejie Li, Ying Wang, Hong Chen, Lehua Yu, Dandong Wu

**Affiliations:** 1 Department of Rehabilitation, The Second Affiliated Hospital of Chongqing Medical University, 76 Linjiang Road, Yuzhong District, Chongqing, 400010 China; 2 Department of Orthopedics, The First Affiliated Hospital of Chongqing Medical University, Chongqing, China

**Keywords:** CTRP4, Leptin signaling, Microglial activation, NF-κB signaling, Diet-induced obesity

## Abstract

**Objective:**

C1qTNF-related protein 4 (CTRP4) acts in the hypothalamus to modulate food intake in diet-induced obese mice and has been shown to exert an anti-inflammatory effect on macrophages. Since high-fat diet-induced microglial activation and hypothalamic inflammation impair leptin signaling and increase food intake, we aimed to explore the potential connection between the anorexigenic effect of CTRP4 and the suppression of hypothalamic inflammation in mice with DIO.

**Methods:**

Using an adenovirus-mediated hypothalamic CTRP4 overexpression model, we investigated the impact of CTRP4 on food intake and the hypothalamic leptin signaling pathway in diet-induced obese mice. Furthermore, central and plasma proinflammatory cytokines, including TNF-α and IL-6, were measured by Western blotting and ELISA. Changes in the hypothalamic NF-κB signaling cascade and microglial activation were also examined in vivo. In addition, NF-κB signaling and proinflammatory factors were investigated in BV-2 cells after CTRP4 intervention.

**Results:**

We found that food intake was decreased, while leptin signaling was significantly improved in mice with DIO after CTRP4 overexpression. Central and peripheral TNF-α and IL-6 levels were reduced by central Ad-CTRP4 administration. Hypothalamic NF-κB signaling and microglial activation were also significantly suppressed in vivo. In addition, NF-κB signaling was inhibited in BV-2 cells following CTRP4 intervention, which was consistent with the decreased production of TNF-α and IL-6.

**Conclusions:**

Our data indicate that CTRP4 reverses leptin resistance by inhibiting NF-κB-dependent microglial activation and hypothalamic inflammation.

**Supplementary Information:**

The online version contains supplementary material available at 10.1186/s12974-021-02167-2.

## Background

In anorexigenic proopiomelanocortin/cocaine- and amphetamine-regulated transcript (POMC/CART) neurons and orexigenic agouti-related peptide/neuropeptide Y (AgRP/NPY) neurons, the hypothalamic leptin signaling pathway plays a key role in the regulation of food intake and energy balance [1]. Leptin signaling is often impaired by high-fat diet (HFD)-induced hypothalamic inflammation [2, 3]. Although metabolic inflammation in the medial basal hypothalamus (MBH) is characterized by the accumulation of astrocytes and microglia, the latest research showed that microglia are essential players in the saturated fat diet-induced inflammatory process [4]. Even during the early course of HFD feeding, microglial recruitment, proliferation, and size changes occurred in conjunction with altered morphology [5]. Moreover, activated microglia induce neurotoxic effects by producing inflammatory cytokines, including tumor necrosis factor-α (TNF-α), interleukin-6 (IL-6), and interleukin-1β (IL-1β) [6, 7]. In addition, several inflammatory pathways were shown to be upregulated [8], including the classic proinflammatory IκBα (inhibitor of κB) kinase β/nuclear factor kappa B (IKKβ/NF-κB) signaling pathway [8]. More interestingly, studies have shown that inhibiting the NF-κB cascade in microglia reduces microgliosis and diet-induced weight gain [4, 9] and restores hypothalamic leptin sensitivity [10], suggesting a promising therapeutic target for HFD-induced obesity.

The CTRP (also referred to as C1qTNF-related protein) family, which features a highly conserved C-terminal complement C1q domain [11], belongs to the adipokine family [12]. It plays important roles in multiple physiological processes, such as inflammation and metabolism [13, 14]. For example, CTRP1 plays an essential role in linking dysregulated lipid metabolism and inflammatory responses in macrophages [15]. Among the CTRP family, CTRP4 is unique in possessing two C1q domains connected by a short linker and acts in the hypothalamus to modulate food intake [16]. It has been demonstrated that central injection of CTRP4 could decrease food intake in mice with diet-induced obesity (DIO); however, the underlying mechanism remains largely unknown [16]. Given that CTRP4 is able to suppress lipopolysaccharide (LPS)-induced proinflammatory cytokine production in macrophages by reducing NF-κB and signal transducer and activator of transcription 3 (STAT3) activation [17], there may be a connection between the anorexigenic effect of CTRP4 and hypothalamic inflammation in mice with DIO.

In the present study, we aimed to investigate whether CTRP4 regulated food intake in mice with DIO by suppressing hypothalamic inflammation. We examined whether CTRP4 exerted its anti-inflammatory effects by attenuating microglial activation and inhibiting the NF-κB pathway, which ultimately led to improved leptin signaling and reduced food intake.

## Methods

### Construction and amplification of recombinant adenoviruses

Recombinant adenoviruses were generated by using Ad-Easy technology as previously described [18]. Full fragments of murine C1qtnf4 (NM_026161.3) were PCR-amplified and subcloned into pAdTrack-CMV. The resultant shuttle vectors were used to generate recombinant adenoviral vectors through homologous recombination with the adenoviral backbone vector in bacterial BJ5183 cells. Subsequently, the recombinant adenovirus Ad-CTRP4 was generated in HEK-293 cells. The resulting Ad-CTRP4 adenoviruses coexpressed GFP as a marker to track infection efficiency.

### Animals

A total of 130 C57BL/6 male mice (6~8 weeks of age; 18~23 g) were used in this study. All mice were housed in temperature-controlled polycarbonate cages (Animal Center of Chongqing Medical University, Chongqing, China) with unlimited access to food and water throughout the study period. During the first week, all mice were fed a standard chow diet (SCD, 10% kcal derived from fat). One week later, the mice were randomly divided into SCD-fed group or high-fat diet (HFD, 60% kcal derived from fat, catalog no. D12492, Research Diets, USA)-fed group. The mice received SCD or HFD for another 20 weeks before the experiments. All animal protocols were approved by the Animal Experimentation Ethics Committee of Chongqing Medical University.

### Stereotaxic cannulation

As previously described, the mice were anesthetized and fixed in a stereotactic apparatus. A unilateral 26-gage guide cannula was implanted into the third ventricle. The stereotaxic coordinates were 0.83 mm anterior and 0 mm right lateral to the bregma and 4.8 mm ventral to the skull. After the animals recovered from surgery (when the body weight of the mice recovered to 90% of their preoperative weight, about 7 days), baseline food intake (for 24 h) was measured. SCD-fed mice were treated i.c.v. with saline (3 μL) (SCD-saline (SCS)) as the control group (n = 10). HFD-fed mice were randomized into the following three groups: (1) HFD-saline (HFS) (n = 10), (2) HFD-Ad-GFP (HFG) (n = 10), (3) HFD-Ad-CTRP4 (HFC) (n = 10). The mice were treated i.c.v. with Ad-CTRP4 (3 μL, 10^10^ pfu), Ad-GFP (3 μL, 10^10^ pfu), or saline (3 μL) at a flow rate of 0.1 μL/min. Seventy-two hours after i.c.v. intervention [18], the mice were anesthetized, plasma and tissue samples were collected for further molecular analysis. In vivo experiments were performed without blinding of the investigators.

### Leptin sensitivity assay

To study the changes of leptin sensitivity, another set of experiment was performed. Sixty hours after central intervention, mice were fasted for 12 h, then either vehicle (saline) or leptin (3 μg/mice) was i.c.v. injected [19]. Fifteen minutes later, the mice were anesthetized, and tissues were collected for further analysis.

### Seven-day-phase food intake in conscious mice

NCD- and HFD-fed mice were individually housed in their familiar housing cages and fed ad libitum. Before the start of the dark cycle, each mouse received one-time i.c.v. injection. NCD-fed mice were infused with saline (3 μL) and HFD-fed mice were infused with saline (3 μL), Ad-GFP (3 μL, 10^10^ pfu), or Ad-CTRP4 (3 μL, 10^10^ pfu). Food intake and body weight were monitored every 24 h for 7 days.

### Determination of plasma parameters

Seventy-two hours after i.c.v. intervention, fasting blood samples were collected retro-orbitally. Plasma was separated by centrifugation and stored at −80 °C for plasma parameter analysis. Serum levels of total cholesterol (TC), triglycerides (TG), glycosylated serum protein (GSP), high-density lipoprotein cholesterol (HDL-c), and low-density lipoprotein cholesterol (LDL-c) were measured with enzymatic colorimetric kits. Plasma glucose was measured by the glucose oxidase method. Plasma TNF-α and IL-6 were measured by ELISA kits (No. 88-7324 and No. 88-7064, Invitrogen, Carlsbad, CA, USA).

### Cell culture and treatments

BV-2 microglial cells (Zhong Qiao Xin Zhou Biotechnology Co., Ltd., Shanghai, China) were cultured in Dulbecco’s modified Eagle’s medium (DMEM) (D6046, Sigma-Aldrich, St. Louis, MO, USA) supplemented with 10% heat-inactivated fetal bovine serum (FBS) and 1% penicillin-streptomycin. Cells were maintained at 37 °C in a humidified 5% CO_2_ incubator. BV-2 microglia were grown to 70% confluence before treatment. To determine the appropriate concentration of palmitate, the cells were treated by replacing the media with fresh media containing 50 μM, 100 μM, or 200 μM palmitate (PA) (N0830, MCE, NJ, USA) or dimethyl sulfoxide (DMSO). Six groups were set as follows: control group, CTRP4 2.5 group, PA group, PA+CTRP4 2.5 group, PA+CTRP4 5 group, and PA+CTRP4 10 group. Recombinant murine CTRP4 protein (2137-TN-050, Novus Biologicals, CO, USA) was diluted to 2.5 μg/ml, 5 μg/ml, and 10 μg/ml in DMEM. Each cell culture plate was preincubated with different concentrations of recombinant murine CTRP4 protein for 2 h as indicated in the figure legend. After 2 h of pretreatment, the cells were treated with 100 μM PA for 24 h before analysis.

### Immunofluorescence analysis

Seventy-two hours after i.c.v. Ad-CTRP4, Ad-GFP, or saline injection, the mice were anesthetized by 50mg/kg sodium pentobarbital (i.p.) and perfused first with saline containing 20 units/mL heparin for 3 min and then with 4% paraformaldehyde in 0.1 mol/L PBS for 20 min, as previously described [18]. Then, the hypothalamus was separated, embedded in optimal cutting temperature compound, immediately frozen on dry ice, and stored at −80 °C. In vitro, BV-2 microglial cells were seeded onto glass coverslips. The samples were fixed in 4% paraformaldehyde, permeabilized with 0.1% Triton X-100, and incubated with a 1:500 dilution of primary antibodies against ionized calcium-binding adapter molecule 1 (Iba1) (Wako Pure Chemical Industries, Ltd., Japan), phospho-P65 (Ser536) (3033, Cell Signaling Technology, Beverly, MA, USA), P65 (8242, Cell Signaling Technology, Beverly, MA, USA), NPY (11976, Cell Signaling Technology, Beverly, MA, USA), POMC (ab210605, Abcam, Cambridge, MA, USA), and CD68 (53444, Abcam, Cambridge, MA, USA). Then, the sections were incubated in fluorescent-conjugated secondary antibodies: goat anti-mouse IgG (DyLight 549, Abbkine, Wuhan, China) and goat anti-rabbit IgG (DyLight 488, Abbkine, Wuhan, China). DNA was stained with 4′,6-diamidino-2-phenylindole (DAPI). Images were captured with a Nikon TE2000U microscope. For Iba1, POMC, NPY, p-P65, P65, and Iba1/CD68 immunostaining in which discrete cells could be identified, cell number was counted manually using ImageJ. For microglia cell size (using Iba1), thresholding was performed in ImageJ, followed by densitometric quantification [2].

### Western blot analysis

Seventy-two hours after i.c.v. Ad-CTRP4, Ad-GFP, or saline injection, samples were harvested from the mice. The hypothalamus was dissected as previously described [18]. Hypothalamus tissues were homogenized. Total protein was extracted using radioimmunoprecipitation assay lysis buffer. Nuclear and cytoplasmic fractions were isolated using an Ne-PER nuclear and cytoplasmic extraction reagent kit (Thermo Scientific, MA, USA). The proteins were extracted in lysis buffer, subjected to 8% SDS-PAGE and transferred to polyvinylidene difluoride membranes. The membranes were probed at 4 °C in the presence of primary antibodies against phospho-P65 (Ser536) (3033, Cell Signaling Technology, Beverly, MA, USA), P65 (8242, Cell Signaling Technology, Beverly, MA, USA), phospho-IKKα/β (Ser176/180) (2697, Cell Signaling Technology, Beverly, MA, USA), IKKα/β (2678, Cell Signaling Technology, Beverly, MA, USA), phospho-IκBα (Ser32/36) (9246, Cell Signaling Technology, Beverly, MA, USA), CTRP4 (36871, Abcam, Cambridge, MA, USA), POMC (210605, Abcam, Cambridge, MA, USA), phospho-Janus kinase 2 (JAK2) (32101, Abcam, Cambridge, MA, USA), phospho-STAT3 (Tyr705) (9145, Cell Signaling Technology, Beverly, MA, USA), STAT3 (9139, Cell Signaling Technology, Beverly, MA, USA), suppressor of cytokine signaling 3 (SOCS3) (2932, Cell Signaling Technology, Beverly, MA, USA), TNF-α (11948, Cell Signaling Technology, Beverly, MA, USA), IL-6 (12912, Cell Signaling Technology, Beverly, MA, USA), NPY (11976, Cell Signaling Technology, Beverly, MA, USA), CD11b (66519-1-1g, Proteintech, Bath, UK), Lamin B1 (12987-1, proteintech, Suite Rosemont, IL, USA), and CD68 (53444, Abcam, Cambridge, MA, USA). After being washed three times with Tris-buffered saline containing 0.1% Tween-20, the membranes were incubated with secondary antibodies for 1 h at room temperature. Next, the blots were visualized with a Bio Imaging System Densitometer (Bio-Rad, Hercules, CA), and quantification of antigen-antibody complexes was performed with Quantity One analysis software (Bio-Rad).

### Statistical analysis

By using PASS 15.0 (NCSS, Utah, USA), the sample size was calculated after analyzing data from our pilot study. To detect differences in food intake at the third day after i.c.v. injection and molecular analysis among groups with a two-tailed α error of 5%, a β error of 20%, ≥5 animals per group were required. The data are presented as the mean ± standard deviation (SD). Statistical analyses were performed using SPSS 23.0 (SPSS, Chicago, IL). A two-tailed unpaired Student’s t test or the Mann-Whitney test, as appropriate, was used for two-group comparisons. One-way or two-way analysis of variance (ANOVA) and Tukey’s test for multiple comparisons were used to analyze differences between groups. A value of P<0.05 was considered significant.

## Results

### Effects of hypothalamic CTRP4 overexpression on energy homeostasis and body metabolism

**Fig. 1:**
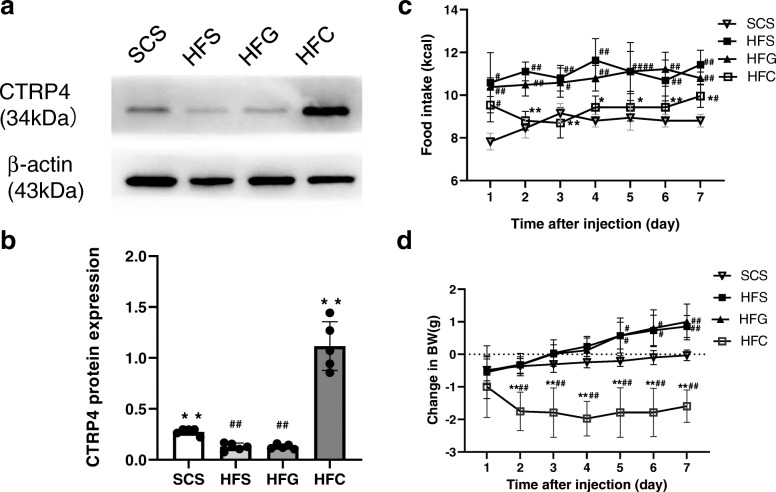
Effects of central Ad-CTRP4 injection on hypothalamic CTRP4 protein expression, food intake, and body weight in mice. (**a**-**b**) At 72 h after i.c.v. injection, hypothalami were collected from mice fed a standard chow diet (SCD) or a high-fat diet (HFD). Representative Western blot (**a**) and quantitative analysis of CTRP4 expression (**b**) in the hypothalamus of mice that received saline (SCS and HFS), Ad-GFP (HFG), and Ad-CTRP4 (HFC). (**c**) Daily food intake after central intervention. (**d**) Changes in body weight after central intervention. The data are the mean ± SD. n = 5 per group. Two-way analysis of variance (ANOVA) and Tukey’s test for multiple comparisons were used to analyze differences among groups. ^#^P<0.05, ^##^P<0.01 vs. SCS group, *P<0.05, **P<0.01 vs. HFG group

Twenty weeks of HFD consumption significantly decreased hypothalamic CTRP4 expression. Hypothalamic CTRP4 protein levels were increased (P<0.05) in HFD-fed mice after i.c.v. Ad-CTRP4 injection compared with the controls (Fig. 1a-b). Then, we explored the effects of CTRP4 on energy homeostasis and revealed that central Ad-CTRP4 intervention caused significant decreases in food intake in HFD-fed mice between days 2 and 7 (Fig. 1c). While there was no significant difference in total body weight between the HFD-fed groups due to the short intervention period (data not shown), i.c.v. Ad-CTRP4 injection significantly slowed the daily increase in body weight of HFD-fed mice (Fig. 1d). We also examined the effects of central CTRP4 on peripheral metabolism and found that the levels of fasting plasma glucose, TG, LDL-c, HDL-c, and GSP were not affected by CTRP4 intervention (Table 1). However, TC levels were significantly decreased in mice that received Ad-CTRP4 injection (Table 1).

**Table 1:** General characteristics of the different groups of C57BL/6J mice

	SCS	HFS	HFG	HFC
FBG (mmol/L)	5.905 ± 0.591	6.788 ± 1.008	6.955 ± 0.391	6.828 ± 0.636
GSP (mmol/L)	5.865 ± 0.490	6.953 ± 0.536^#^	6.897 ± 0.457^#^	6.849 ± 0.644^#^
TG (mmol/L)	1.389 ± 0.094	1.834 ± 1.969^##^	1.773 ± 0.157^##^	1.713 ± 0.165^#^
TC (mmol/L)	4.187 ± 0.189	6.258 ± 0.529^##^	6.157 ± 0.621^##^	5.107 ± 0.304^#^**
HDL-c (mmol/L)	0.536 ± 0.664	1.117 ± 0.109^##^	1.006 ± 0.258^##^	0.995 ± 0.210^##^
LDL-c (mmol/L)	2.044 ± 0.127	2.876 ± 0.226^##^	2.707 ± 0.314^##^	2.821 ± 0.118^##^

*FBG* fasting blood glucose, *GSP* glycosylated serum protein, *TG* triglyceride, *TC* total cholesterol, *HDL-c* high-density lipoprotein cholesterol, *LDL-c* low-density lipoprotein cholesterol. The data are the mean ± SD; n = 7

^#^P < 0.05

^##^P < 0.01 vs. the SCS group

**P < 0.01 vs. the HFG group

### Effects of central CTRP4 overexpression on hypothalamic leptin signaling

**Fig. 2:**
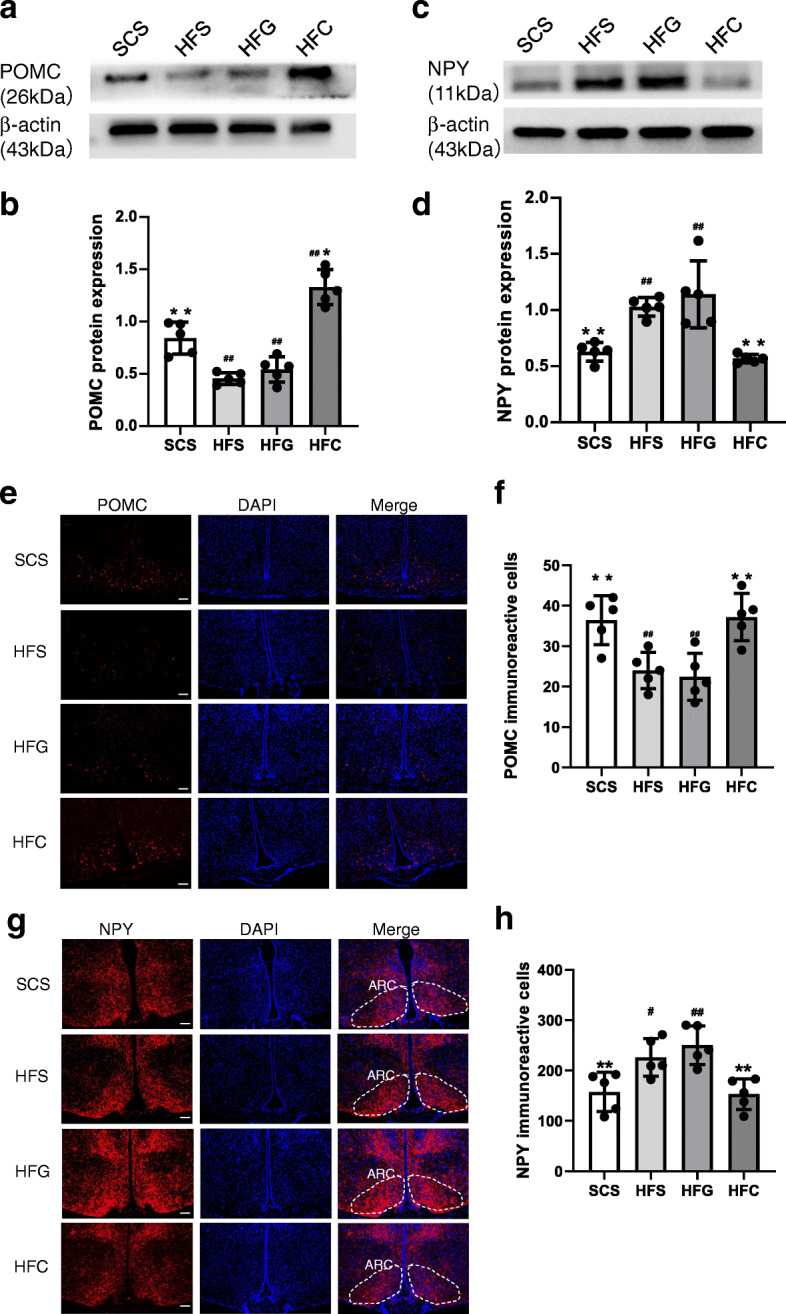
Effects of central CTRP4 overexpression on hypothalamic neuropeptides by Western blot and immunofluorescence analysis. At 72 h after i.c.v. injection, the mice receiving saline (SCS and HFS), Ad-GFP (HFG), and Ad-CTRP4 (HFC) were sacrificed, and then the hypothalami were dissected. (**a**-**d**) Representative Western blot and quantitative analysis of POMC (**a**-**b**) and NPY (**c**-**d**) in hypothalami. (**e**) Representative images of POMC immunoreactivity (red) and DAPI nuclear staining (blue) in the ARCs of mice among groups. (**f**) Densitometric analysis of POMC-immunoreactive cells among groups. (**g**) Representative images of NPY immunoreactivity (red) and DAPI nuclear staining (blue) in the ARCs of mice among groups. (**h**) Densitometric analysis of NPY-immunoreactive cells among groups. n = 5 per group. Scale bar = 25μm. Two-way analysis of variance (ANOVA) and Tukey’s test for multiple comparisons were used to analyze differences among groups. ^#^P<0.05, ^##^P<0.01 vs. SCS group, *P<0.05, **P<0.01 vs. HFG group

**Fig. 3:**
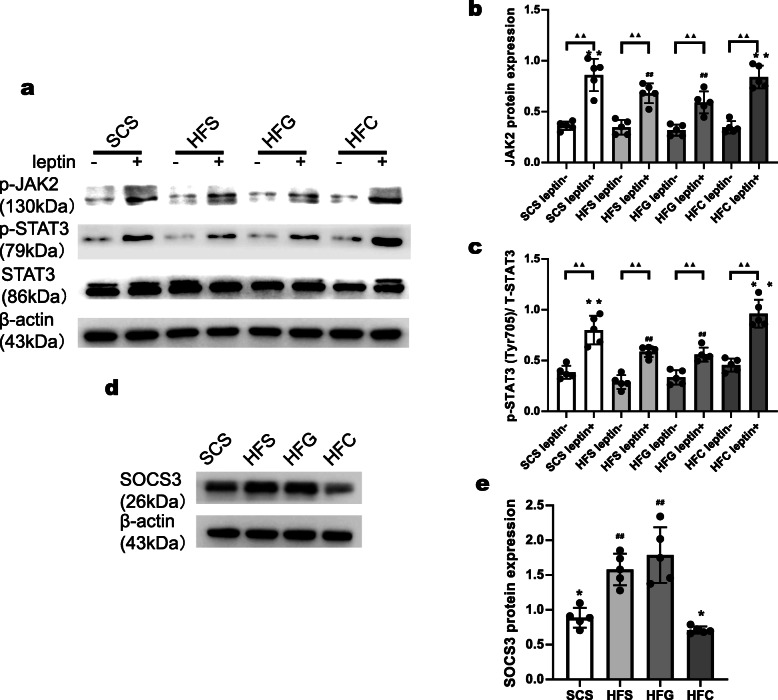
Effects of central CTRP4 overexpression on hypothalamic leptin signaling. (**a**-**c**) 60 h after central intervention, mice were fasted for 12 h, then either saline (leptin− group) or leptin (3 μg/mice) (leptin+ group) was i.c.v. injected. Fifteen minutes later, the mice were anesthetized, and hypothalamic tissue was collected for Western blot analysis. (**a**) Immunoblots showing p-STAT3^Tyr705^, STAT3, p-JAK2, and β-actin levels in saline- and leptin-stimulated mice fed an SCD (SCS) or HFD (HFS, HFG, and HFC) after central intervention. (**b**-**c**) Quantitative analysis of p-STAT3^Tyr705^ and p-JAK2 expression. (**d**-**e**) At 72 h after i.c.v. injection, the mice were anesthetized, and then hypothalamic tissue was collected for SOCS3 protein analysis. (**d**) Representative immunoblots showing hypothalamic SOCS3 in SCD- and HFD-fed mice that received saline (SCS and HFS), Ad-GFP (HFG), and Ad-CTRP4 (HFC). (**e**) Quantitative analysis of SOCS3 expression. n = 5 per group. A two-tailed unpaired Student’s t test or the Mann-Whitney test, as appropriate, was used for two-group comparisons. Two-way analysis of variance (ANOVA) and Tukey’s test for multiple comparisons were used to analyze differences among groups. (**b**-**c**) ^▲^P<0.05, ^▲▲^P<0.01 leptin+ group vs. leptin− group, ^#^P<0.05, ^##^P<0.01 vs. SCS leptin+ group, *P<0.05, **P<0.01 vs. HFG leptin+ group. (**e**) ^#^P<0.05, ^##^P<0.01 vs. SCS group, *P<0.05, **P<0.01 vs. HFG group

We investigated the mechanism by which CTRP4 suppressed food intake and found that POMC expression was obviously increased by CTRP4 intervention in the HFD-fed group (Fig. 2a-b), while NPY expression was significantly decreased (Fig. 2c-d). To determine the effect of central Ad-CTRP4 on POMC- and NPY-expressing neurons, we also performed immunofluorescence analysis in mouse hypothalamic tissue. As shown in Fig. 2e-h, CTRP4 overexpression led to a significant increase in the number of POMC-positive cells but a decrease in the number of NPY-positive cells in the ARC. Then, we examined whether central CTRP4 treatment could improve hypothalamic leptin sensitivity in mice with DIO. In standard chow diet-fed mice, there was a rapid and dramatic increase in the phosphorylation of STAT3 and JAK2 following central leptin injection. However, 20 weeks of HFD feeding significantly blunted this response, while central CTRP4 injection completely rescued leptin-induced STAT3 and JAK2 phosphorylation (Fig. 3a-c). In addition, there was a dramatic decrease in the expression of SOCS3 (Fig. 3d-e), an inhibitor of leptin signaling, suggesting that CTRP4 restored leptin sensitivity by inhibiting SOCS3 expression.

### Effects of central CTRP4 overexpression on microglial activation and hypothalamic and peripheral inflammation

**Fig. 4:**
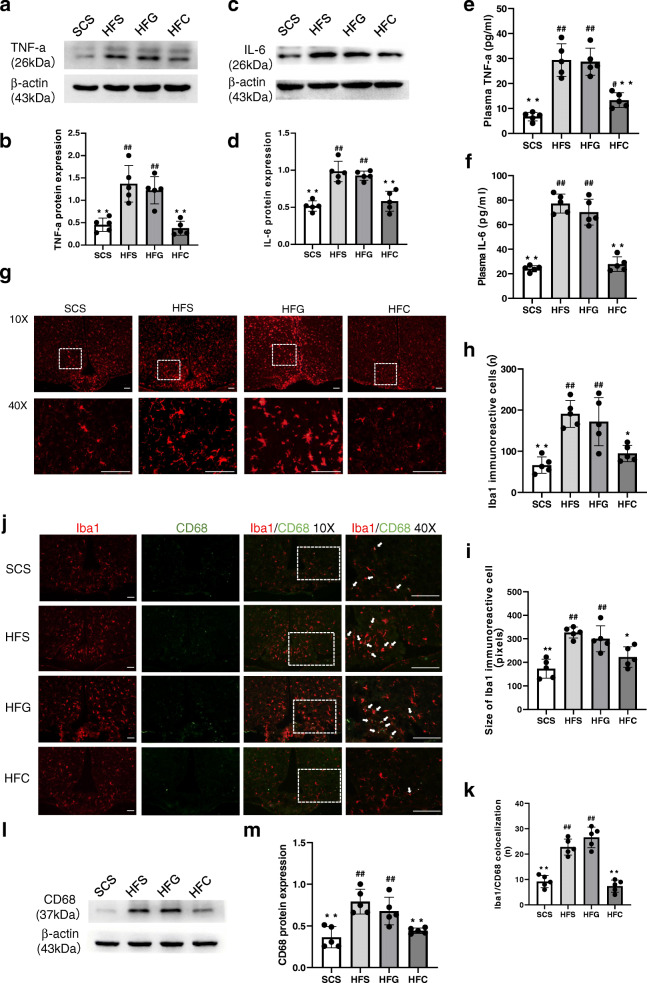
Effects of central CTRP4 overexpression on inflammatory factors and microglial activation. At 72 h after i.c.v. injection, the mice that received saline (SCS and HFS), Ad-GFP (HFG), and Ad-CTRP4 (HFC) were anesthetized, hypothalamic tissue and blood samples were collected. (**a**-**d**) Representative immunoblots and quantitative analysis showing hypothalamic TNF-α (**a**-**b**) and IL-6 (**c**-**d**) in mice among groups. (**e**-**f**) Plasma TNF-α (**e**) and IL-6 (**f**) levels were measured by ELISA. (**g**) Representative images of Iba1 immunoreactivity in the ARCs of mice after saline (SCS and HFS), Ad-GFP (HFG), or Ad-CTRP4 intervention (HFC). Mean ARC microglial cell number (**h**) (per field defined in **g**) and microglia cell size (**i**) (average number of pixels in 10 largest cells) among the different groups. (**j**) Immunofluorescence double labeling for CD68(green), Iba1 (red), and colocalization of Iba1/CD68 are shown. (**k**) Quantitative analysis of the results in panel **j**. (**l**-**m**) Representative Western blot (**l**) and quantitative analysis of CD68 expression in hypothalami. n = 5 per group. Scale bar = 25μm. Two-way analysis of variance (ANOVA) and Tukey’s test for multiple comparisons were used to analyze differences among groups. ^#^P<0.05, ^##^P<0.01 vs. SCS group, *P<0.05, **P<0.01 vs. HFG group

We then examined whether the improved leptin signaling sensitivity was associated with decreased central inflammation. We measured the expression of hypothalamic TNF-α and IL-6 after i.c.v. Ad-CTRP4 intervention. As shown in Fig. 4a-d, the protein levels of IL-6 and TNF-α decreased significantly in the hypothalamus after central Ad-CTRP4 intervention. In addition, plasma TNF-α and IL-6 levels also decreased significantly after central Ad-CTRP4 injection (Fig. 4e-f). Since microglia are the main source of inflammatory cytokines [20], we further evaluated microglial activation in the arcuate nucleus (ARC). Ibal immunostaining revealed that microglial number increased in the ARC of HFD-fed mice, compared with that in SCD-fed controls (Fig. 4g, 4h). Concomitantly, the microglial cell presented with an enlarged and more activated morphology (Fig. 4g, 4i). As shown in Fig. 4g-i, microglial number and cell size decreased after central Ad-CTRP4 injection. Besides, there was a significant increase in the number of Iba1/CD68 colocalized microglia in the ARC (Fig. 4j-k) following HFD-consumption, which was accompanied with increased CD68 protein expression (Fig. 4l-m). After i.c.v. Ad-CTRP4 injection, both Iba1/CD68 colocalization and CD68 protein levels tended to be less (Fig. 4j-m), suggesting that CTRP4 overexpression blunted HFD-induced microglial activation [21].

### Effects of central CTRP4 treatment on NF-κB signaling

**Fig. 5:**
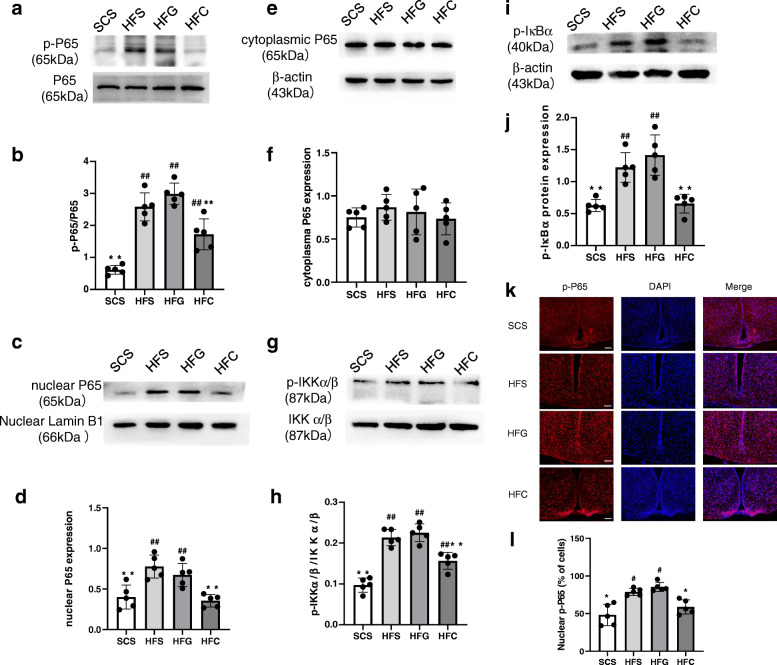
Effects of central CTRP4 overexpression on hypothalamic NF-κB signaling. At 72 h after i.c.v. injection, the mice receiving saline (SCS and HFS), Ad-GFP (HFG), and Ad-CTRP4 (HFC) were anesthetized and hypothalamic tissue was collected. (**a**, **c**, **e**, **g**, **i**) Representative immunoblots showing hypothalamic total p-P65 and P65 (**a**), nuclear and cytoplasmic P65 (**c**, **e**), p-IKKα/β and IKKα/β (**g**), and p-IκBα (**i**) in mice among groups. (**b**, **d**, **f**, **h**, **j**) Quantitative analysis of p-P65 (**b**), nuclear and cytoplasmic P65 (**d**, **f**), p-IKKα/β (**h**) and p-IκBα (**j**) protein. (**k**) Representative images of p-P65 immunoreactivity (red) and DAPI nuclear staining (blue) in the ARCs of mice among groups. (**l**) Quantitative analysis of the results in panel **k**. n = 5 per group. Two-way analysis of variance (ANOVA) and Tukey’s test for multiple comparisons were used to analyze differences among groups. ^#^P<0.05, ^##^P<0.01 vs. SCS group, *P<0.05, **P<0.01 vs. HFG group

As NF-κB signaling is a crucial inflammatory pathway, we next assessed the effects of central CTRP4 on the phosphorylation of P65, inhibitor of NF-κB kinase subunit α/β (IKKα/β) and IκBα, which are proteins in the NF-κB signaling cascade, by Western blotting. The phosphorylation of P65 (Fig. 5a-b), IKKα/β (Fig. 5g-h), and IκBα (Fig. 5i-j) was dramatically increased by HFD consumption and was inhibited by CTRP4 intervention. Immunofluorescence also clearly demonstrated that there was a significant increase in the number of p-P65 staining in the ARC following HFD consumption, which was attenuated by i.c.v. CTRP4 intervention (Fig. 5k-l). Moreover, Western blot further showed that HFD consumption promoted nuclear translocation of NF-κB P65 from the cytosol to the nucleus, while CTRP4 partly abolished NF-κB nuclear translocation (Fig. 5c-f).

### Effects of CTRP4 on NF-κB signaling in palmitate-activated microglia

**Fig. 6:**
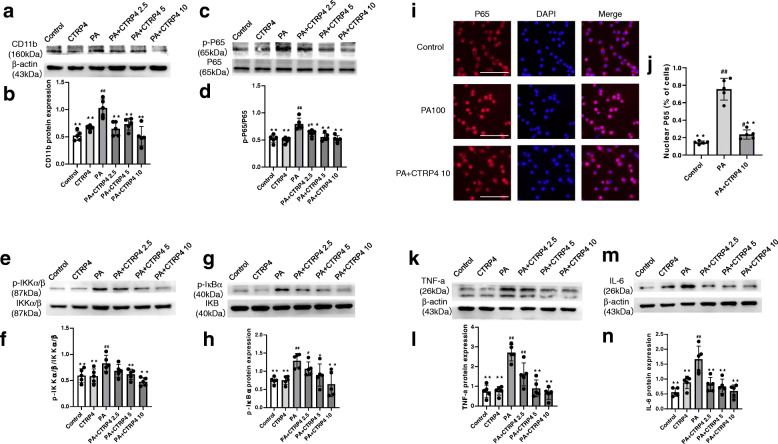
Effects of central recombinant CTRP4 on NF-κB signaling, proinflammatory factors, and microglial activation (BV-2 cells). BV-2 cells were pretreated with DMEM or 2.5 μg/ml, 5 μg/ml, or 10 μg/ml recombinant murine CTRP4 protein for 2 h, followed by 100 μM palmitate or DMSO for 24 h before further analysis. (**a**, **c**, **e**, **g**, **k**, **m**) Representative immunoblots showing CD11b (**a**), p-P65 and P65 (**c**), p-IKKα/β and IKKα/β (**e**), p-IκBα (**g**), TNF-α (**k**), and IL-6 (**m**) in BV-2 cells. (**b**, **d**, **f**, **h**, **l**, **n**) Quantitative analysis of CD11b (**b**), p-P65 (**d**), p-IKKα/β (**f**), p-IκBα (**h**), TNF-α (**l**), and IL-6 (**n**) protein. (**i**-**j**) BV-2 cells were pretreated with 10 μg/ml recombinant murine CTRP4 protein or DMEM for 2 h, followed by 100 μM palmitate for 24 h before immunofluorescence analysis. (**i**) Representative images of P65 immunoreactivity (red) and DAPI nuclear staining (blue) in BV-2 cells. (**j**) Quantitative analysis of the results in panel **i**. n = 5 per group. One-way analysis of variance (ANOVA) and Tukey’s test for multiple comparisons were used to analyze differences among groups. ^#^P<0.05, ^##^P<0.01 vs. the control group, *P<0.05, **P<0.01 vs. PA group

To determine the appropriate palmitate concentration to activate microglia, we assessed CD11b expression in BV-2 cells treated with different concentrations of palmitate (50, 100, and 200 μM). We found that the CD11b protein was most abundant in BV-2 cells exposed to 100 μM PA (Supplementary 1), suggesting that 100 μM was the most appropriate concentration to induce microglial activation. Next, BV-2 cells were pretreated with DMSO or different concentrations of CTRP4 (2.5 μg/ml, 5 μg/ml, and 10 μg/ml) for 2 h, followed by DMSO or palmitate (100 μM) treatment for an additional 24 h. Recombinant CTRP4 protein dose-dependently suppressed palmitate-induced CD11b expression (Fig. 6a-b). Moreover, Western blotting showed that CTRP4 reduced PA-induced phosphorylation of P65, IKKα/β, and IκBα, suggesting that the NF-κB signaling cascade was suppressed by CTRP4 intervention (Fig. 6c-h). Next, we investigated the nuclear translocation of P65, which is a critical step in the NF-κB signaling cascade. In the resting state, P65 remained mostly in the cytosol; however, following PA stimulation, P65 was significantly increased in the nucleus. CTRP4 intervention significantly decreased PA-induced P65 nuclear translocation (Fig. 6i-j). We measured the protein expression of proinflammatory cytokines. PA treatment induced the protein expression of TNF-α and IL-6, whereas CTRP4 reduced PA-induced expression of these proinflammatory cytokines (Fig. 6k-n).

## Discussion

The effects of hypothalamic leptin, which mainly occur in the arcuate nucleus, is particularly important in the regulation of energy homeostasis and peripheral metabolism. However, in the context of DIO, leptin resistance develops in the hypothalamus, whereby exogenous leptin cannot phosphorylate STAT3, further disrupting global energy homeostasis. Thus, restoring leptin sensitivity is a topic of interest worldwide. Phenotypically, due to the relatively short observing period [22], the decrease of body weight did not reach statistical significance in the present study. However, hypothalamic CTRP4 overexpression could indeed significantly decrease food intake in HFD-induced obese mice. Mechanistically, central CTRP4 intervention restored the normal response of p-STAT3^tyr705^ to exogenous leptin stimulation in obese mice, which was blunted by a HFD, indicating the re-establishment of leptin sensitivity. Furthermore, the expression of SOCS3, a leptin signaling inhibitor, was suppressed by central CTRP4 administration. Taken together, these data suggested that hypothalamic CTRP4 overexpression could improve central leptin signaling and energy homeostasis in mice with DIO.

We further examined the effect of CTRP4 on hypothalamic inflammation, and our results demonstrated that central (hypothalamus) and peripheral (plasma) TNF-α and IL-6 levels were reduced by central Ad-CTRP4 intervention. However, the roles of these cytokines in central inflammation differ to some extent. TNF-α is a classic proinflammatory factor that causes hypothalamic insulin and leptin resistance and is believed to be a contributor to the development of obesity [23–25]. On the other hand, the pro- or anti-inflammatory role of IL-6 is thought to be context-dependent [26]. However, a recent review suggested that IL-6 could induce marked microglial activation and drive hypothalamic neuroinflammation, indicating that IL-6 is a proinflammatory cytokine in the central nervous system [27]. Peripherally, TNF-α and IL-6 are both thought to be proinflammatory cytokines. Previous studies have demonstrated that these factors could be downregulated in the hypothalamus via vagus nerve activation [28]. Acetylcholine, the main neurotransmitter of the vagus nerve, inhibits the production of proinflammatory cytokines through the nicotinic receptor in macrophages [29, 30]. Interestingly, the excitability of the vagus nerve is decreased in diet-induced obese rodent models [31]. Thus, central CTRP4 overexpression inhibits hypothalamic inflammation and possibly exerts systemic anti-inflammatory effects through vagus nerve stimulation.

Chronic HFD feeding leads to low-grade activation of proinflammatory NF-κB signaling [8, 32, 33]. Previously, neuronal and astrocytic NF-κB signaling received much attention, as deleting or inhibiting NF-κB in neurons or astrocytes mitigated DIO [32, 34]. However, microglial NF-κB signaling has been found to be critical in the onset of DIO, and selectively restraining microglial NF-κB signaling greatly reduced microglial activation and limited diet-induced hyperphagia and weight gain [9]. In the present study, both changes in the quantity and size of Iba1-labeled cells and decreased Iba1/CD68 colocalization suggested that central CTRP4 overexpression inhibited HFD-induced microglial activation. Microglia are essential players responsible for increased inflammatory cytokine expression in high-fat environments and are resident immune cells in the brain [4]. Thus, the CTRP4-induced in vivo TNF-α and IL-6 downregulation observed in our study could be, at least in part, associated with suppressed microglial activation. On the other hand, Western blotting and immunofluorescence results showed that central CTRP4 overexpression blunted the recruitment of the NF-κB pathway by inhibiting the degradation and phosphorylation of IκB and the nuclear translocation of P65 in HFD-fed mice. PA, a common saturated fatty acid in human diets, is accumulated in the hypothalamus after HFD consumption [35]. By using a BV-2 cell model, in vitro data further confirmed that CTRP4 pretreatment could effectively suppress PA-induced NF-κB signaling cascades and microglial activation, accompanied by decreased production of TNF-α and IL-6, which are targets of NF-κB signaling. Since hypothalamic CTRP4 significantly decreased following HFD consumption, the data in vitro suggested that upregulation of central CTRP4 protein could probably resist HFD-induced microglial NF-κB activation and obesity. However, the current study could not determine whether the inhibition of NF-κB signaling only occurred in microglia or in both neuronal and glial cells.

Reactive microgliosis, which is triggered by saturated fatty acids, leads to neuronal injury, and POMC neurons are especially vulnerable to this phenomenon [2, 36–38]. Recent studies have demonstrated that microglia also interfere with nutrient sensing by the hypothalamus, as restraining microglial activation by an NF-κB-dependent method reduces food intake, mitigates DIO and improves leptin signaling [4, 10]. Our data revealed that central CTRP4 intervention restored leptin signaling and decreased food intake, possibly by suppressing NF-κB-dependent microglial activation. However, little is known about the underlying mechanisms [39]. Previous studies suggest the presence of proinflammatory signals or other factors produced by activated microglia that modulate or impair neuronal responsiveness to leptin and regulate energy homeostasis [39, 40]. Among these factors, TNF-α may mediate such microglia-neuronal cross-talk by inducing endoplasmic reticulum stress and inflammatory signaling cascades in appetite-regulating neurons [41]. Therefore, it is possible that CTRP4 acts on metabolism by suppressing microglial activation and TNF-α production. However, other mechanisms might also be involved in this process and contribute to the anti-inflammatory and metabolic effects of CTRP4 and should be the focus of future work.

## Conclusion

Taken together, these results indicate that CTRP4 reverses leptin resistance by inhibiting NF-κB-dependent microglial activation and hypothalamic inflammation. The evidence provided in our study suggests that targeting hypothalamic microglia via adipokines may be a promising way to mitigate diet-induced metabolic dysfunction.

## Supplementary Information

**Supplementary 1.**

## Data Availability

The datasets used and/or analyzed during the current study are available from the corresponding author on reasonable request.

## Abbreviations

POMC: Proopiomelanocortin
CART: Cocaine- and amphetamine-regulated transcript
AgRP: Agouti-related peptide
NPY: Neuropeptide Y
HFD: High-fat diet
MBH: Medial basal hypothalamus
TNF-α: Tumor necrosis factor-α
IL-6: Interleukin-6
IKKβ: IκBα kinase β
IκBα: Inhibitor of κB
NF-κB: Nuclear factor kappa B
CTRP: C1qTNF-related protein
STAT3: Signal transducer and activator of transcription 3
SCD: Standard chow diet
TC: Total cholesterol
TG: Triglycerides
GSP: Glycosylated serum protein
HDL-c: High-density lipoprotein cholesterol
LDL-c: Low-density lipoprotein cholesterol
JAK2: Janus kinase 2
SOCS3: Suppressor of cytokine signaling 3
ARC: Arcuate nucleus
LPS: Lipopolysaccharide
PA: Palmitate
