# Supplementary figures and images for: C1q/TNF-related protein 4 restores leptin sensitivity by downregulating NF-κB signaling and microglial activation
Source: J Neuroinflammation. 2021 Jul 18;18:159. doi: 10.1186/s12974-021-02167-2 (PMC8286609; doi:10.1186/s12974-021-02167-2)

## Slide 1
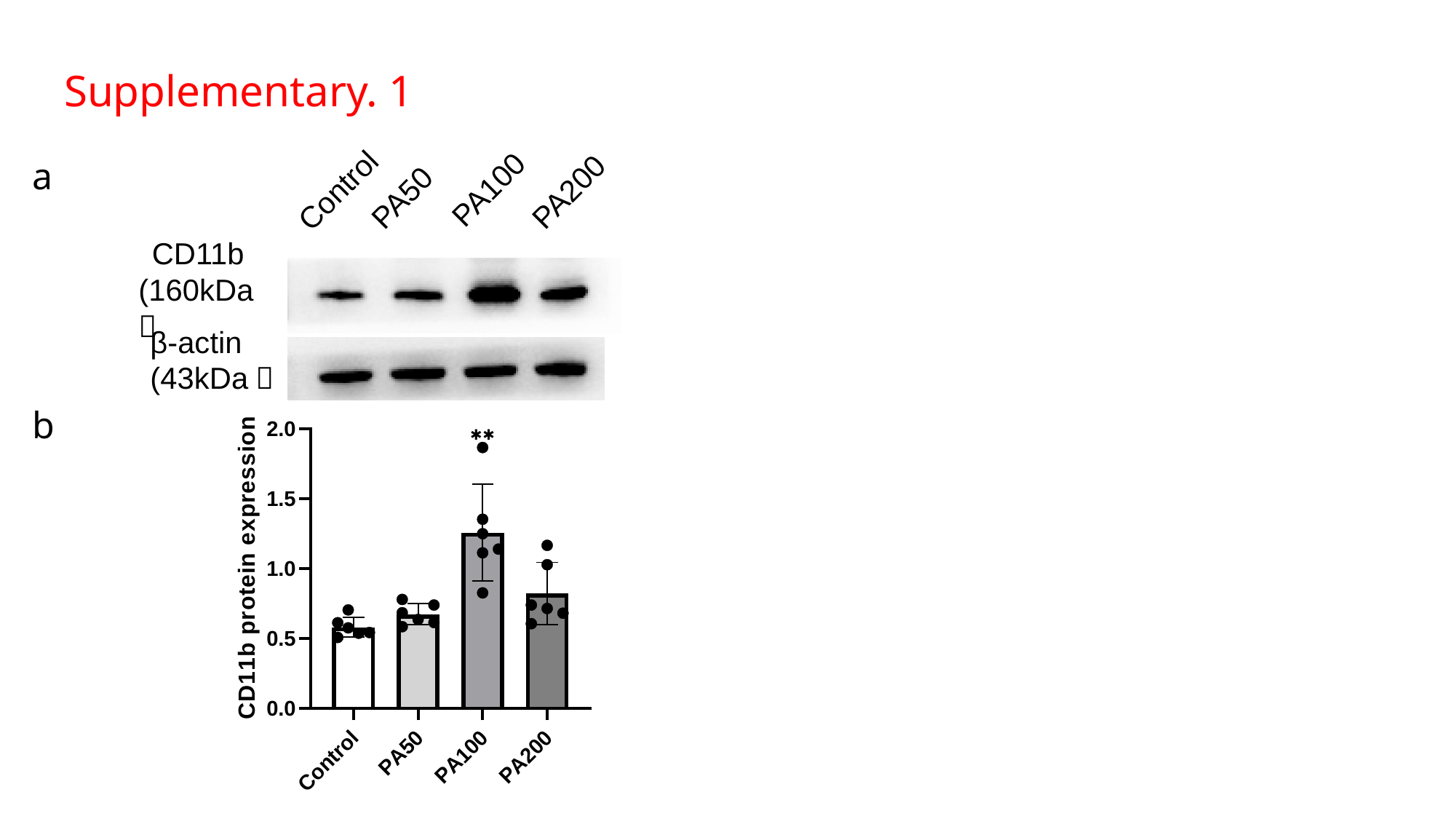

Supplementary. 1
PA200
PA100
a
PA50
Control
CD11b
(160kDa）
β-actin
(43kDa）
b

Supplement: Supplementary file 1 — Supplementary 1. [file 12974_2021_2167_MOESM1_ESM.pptx]
